# Incidence, risk factors, management strategies, and outcomes of antibody-mediated rejection in pediatric kidney transplant recipients—a multicenter analysis of the Cooperative European Paediatric Renal Transplant Initiative (CERTAIN)

**DOI:** 10.1007/s00467-024-06487-2

**Published:** 2024-09-16

**Authors:** Alexander Fichtner, Laura Gauché, Caner Süsal, Thuong Hien Tran, Rüdiger Waldherr, Kai Krupka, Isabella Guzzo, Andrea Carraro, Jun Oh, Matthias Zirngibl, Marcus Weitz, Jens König, Anja Büscher, Laszlo Berta, Thomas Simon, Atif Awan, Krisztina Rusai, Rezan Topaloglu, Licia Peruzzi, Nikoleta Printza, Jon Jin Kim, Lutz T. Weber, Anette Melk, Lars Pape, Susanne Rieger, Christian Patry, Britta Höcker, Burkhard Tönshoff, for the CERTAIN study group

**Affiliations:** 1 Heidelberg University, Medical Faculty Heidelberg, Department of Pediatrics I, University Children’s Hospital, Im Neuenheimer Feld 430, 69120 Heidelberg, Germany; 2 Heidelberg University, Medical Faculty Heidelberg, Institute of Immunology, Transplantation Immunology, Heidelberg, Germany; 3 Transplant Immunology Research Center of Excellence, Koç University, Istanbul, Turkey; 4 Heidelberg University, Medical Faculty Heidelberg, Department of Pathology, Heidelberg, Germany; 5 Pediatric Nephrology and Renal Transplant Unit, Bambino Gesù Children’s Hospital–IRCCS, Rome, Italy; 6 Pediatric Nephrology, Dialysis and Transplantation Unit, Department of Woman’s and Child’s Health, University Hospital of Padova, Padua, Italy; 7 Department of Pediatric Nephrology, University Children’s Hospital, Hamburg, Germany; 8 Department of General Pediatrics and Hematology/Oncology, University Children’s Hospital, University Hospital Tübingen, Tübingen, Germany; 9 Department of General Pediatrics, University Children’s Hospital Münster, Münster, Germany; 10 Clinic for Paediatrics III, Essen University Hospital, Essen, Germany; 11 Pediatric Center, MTA Center of Excellence, Semmelweis University, Budapest, Hungary; 12 Pediatric Nephrology, Toulouse University Hospital, Toulouse, France; 13 Temple Street Children’s University Hospital, Dublin, Ireland; 14 Division of Paediatric Nephrology and Gastroenterology, Department of Paediatrics and Adolescent Medicine, Comprehensive Center for Pediatrics, Medical University of Vienna, Vienna, Austria; 15 Haceteppe University, Ankara, Turkey; 16 Pediatric Nephrology Dialysis and Transplantation Unit, Regina Margherita Children’s Hospital, Turin, Italy; 17 1st Department of Pediatrics, Aristotle University of Thessaloniki, Thessaloniki, Greece; 18 Department of Paediatric Nephrology, Nottingham University Hospital, Nottingham, UK; 19 Pediatric Nephrology, Children’s and Adolescents’ Hospital, Faculty of Medicine and University Hospital of Cologne, University of Cologne, Cologne, Germany; 20 Department of Pediatric Kidney, Liver and Metabolic Diseases, Hannover Medical School, Hannover, Germany

**Keywords:** Antibody-mediated rejection, Pediatric kidney transplantation, Risk factors, *Dn*DSA development, Graft outcome, BKPyV nephropathy

## Abstract

**Background:**

This study by the Cooperative European Paediatric Renal Transplant Initiative (CERTAIN) was designed to determine the incidence, risk factors, current management strategies, and outcomes of antibody-mediated rejection (ABMR) in pediatric kidney transplant recipients (pKTR).

**Methods:**

We performed an international, multicenter, longitudinal cohort study of data reported to the Cooperative European Paediatric Renal Transplant Initiative (CERTAIN) registry. Three hundred thirty-seven pKTR from 21 European centers were analyzed. Clinical outcomes, including kidney dysfunction, rejection, HLA donor-specific antibodies, BK polyomavirus-associated (BKPyV) nephropathy, and allograft loss, were assessed through 5 years post-transplant.

**Results:**

The cumulative incidence of de novo donor-specific class I HLA antibodies (HLA-DSA) post-transplant was 4.5% in year 1, 8.3% in year 3, and 13% in year 5; the corresponding data for de novo class II HLA-DSA were 10%, 22.5%, and 30.6%, respectively. For 5 years post-transplant, the cumulative incidence of acute ABMR was 10% and that of chronic active ABMR was 5.9%. HLA-DR mismatch and de novo HLA-DSA, especially double positivity for class I and class II HLA-DSA, were significant risk factors for ABMR, whereas cytomegalovirus (CMV) IgG negative recipient and CMV IgG negative donor were associated with a lower risk. BKPyV nephropathy was associated with the highest risk of graft dysfunction, followed by ABMR, T-cell mediated rejection, and older donor age.

**Conclusions:**

This study provides an estimate of the incidence of de novo HLA-DSA and ABMR in pKTR and highlights the importance of BKPyV nephropathy as a strong risk factor for allograft dysfunction.

**Graphical abstract:**

**Figure:**
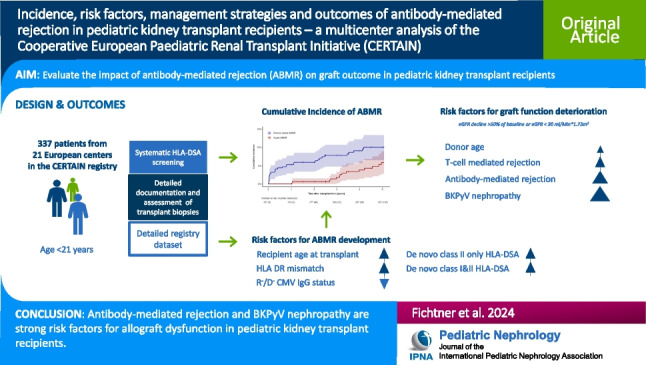
A higher resolution version of the Graphical abstract is available as Supplementary information

**Supplementary Information:**

The online version contains supplementary material available at 10.1007/s00467-024-06487-2.

## Introduction

Kidney transplantation is the treatment of choice for children and adolescents with stage 5 chronic kidney disease (CKD). However, long-term graft survival is variable and dependent on multiple risk factors. In adults, antibody-mediated rejection (ABMR) is recognized as the leading cause of kidney allograft failure. Over the past decade, the pivotal causative role of donor-specific HLA antibodies (HLA-DSA) in ABMR and premature allograft failure has been established [1]. The overall incidence of ABMR in adults is estimated to range from 1 to 21.5%, depending on the definition of ABMR, the cohort studied (i.e., non-sensitized, ABO incompatible, or HLA-sensitized patients), the live vs*.* deceased donor status, and the time period considered post-transplant (early vs. late) [2]. In adult kidney transplant recipients, risk factors for ABMR include pre-formed HLA-DSA due to previous immunization events such as previous transplants, blood transfusions, or pregnancy in female recipients, a high degree of HLA mismatch, especially against HLA class II antigens such as HLA-DR and HLA-DQ, and under-immunosuppression, either due to non-adherence to immunosuppressive medications or physician-initiated tapering of immunosuppressive medications due to side effects or recurrent infections [1–3].

While some of these risk factors also apply to the pediatric patient population, there are pediatric-specific risk factors such as immunologic naivete to transplant-relevant viral infections in young children and poor adherence to immunosuppressive medications in adolescents [4–6]. In addition, children on the transplant waiting list are less likely to be presensitized than adults. Therefore, data on the incidence and outcome of ABMR in adults cannot be readily extrapolated to the pediatric patient population. Currently, there are no robust data available on the epidemiology and outcomes of ABMR in pediatric kidney transplant recipients. Therefore, the aim of the present study was to analyze the incidence, risk factors, management strategies, and outcomes of ABMR in a large cohort of European pediatric kidney transplant recipients.

## Materials and methods

### Study design and patients

We performed an international, multicenter, longitudinal cohort study of data reported to the Cooperative European Paediatric Renal Transplant Initiative (CERTAIN) registry (https://www.certain-registry.eu) [7, 8]. This registry allows an in-depth characterization of specific patient cohorts, due to its detailed and comprehensive data collection. Specific and additional data collection for this analysis was performed according to a defined study protocol. Eligible patients were pediatric kidney allograft recipients (i) aged ≤ 21 years at the time of transplantation, (ii) with a complete and validated data set, (iii) with de novo HLA-DSA surveillance at least once a year, (iv) HLA-DSA measurement at the time of indication biopsy or surveillance (protocol) biopsy of the kidney transplant prior to initiation of therapy. All diagnostic tests for de novo HLA-DSA events had to be documented including those that were negative. Patients received their kidney transplant between October 1, 2006, and September 30, 2021, with a minimum follow-up of 1 year. Data were collected on day 0 before transplantation, at months 1, 3, 6, 9, and 12 post-transplant, and every 6 months thereafter. Written informed consent was obtained from all parents or guardians to participate in the registry, with assent from patients when appropriate for their age. The CERTAIN registry was approved by the ethics committee of each contributing center and fully complies with the principles of the Declaration of Helsinki and Good Clinical Practice guidelines. The study was designed, analyzed, and reported according to the STROBE guidelines (https://www.strobe-statement.org).

### Immunosuppressive therapy

The initial immunosuppressive regimen usually consisted of a calcineurin inhibitor, namely tacrolimus or cyclosporine microemulsion; an antiproliferative agent, namely mycophenolate mofetil and azathioprine; or a mammalian target of rapamycin inhibitor (everolimus) and glucocorticoids. Induction therapy with an interleukin-2 receptor (IL-2R) antagonist or thymoglobulin was performed according to center practice.

### Donor-specific HLA antibodies and BK polyomavirus analysis

Patient sera were tested for HLA antibodies and their specificities with Luminex technology according to center practice, at least once annually and at the time of an indication biopsy. LABScreen Luminex kits from One Lambda (Canoga Park, CA, USA) were used according to the manufacturer’s instructions in 19 of 21 (90.5%) centers; 2 of 21 (9.5%) centers used the LIFECODES assay from Immucor (Georgia, USA). Samples were treated with dithiothreitol (DTT), ethylenediaminetetraacetic acid (EDTA), or heat-treated to control for the prozone effect. Anti-HLA antibody screening was performed on historical sera for the waiting list. De novo HLA-DSA was defined as any HLA-DSA specificities detected post-transplant that were not detectable pretransplant. The mean fluorescence intensity (MFI) cut-off value used to define post-transplant HLA-DSA positivity varied between 500 and 3000 among the 21 participating centers. Irrespective of the individual definition of HLA-DSA positivity in the participating centers, an MFI value of ≥ 1400 was defined as a uniform cut-off value for HLA-DSA positivity in this multicenter setting [9]. The HLA-DSA with the highest MFI value in each serum sample was defined as the immunodominant HLA-DSA. Molecular HLA typing was performed using sequence-specific oligonucleotide analysis at the HLA-A, -B, and DR-loci. Some centers provided additional typing information for HLA-Cw (*n* = 13), HLA-DP (*n* = 5), and HLA-DQ (*n* = 13).

Assessment of BK polyomavirus (BKPyV) DNAemia and BKPyV-associated nephropathy was performed according to center practice. Quantitative nucleic acid testing was performed using commercially available assays [10]. Biopsy-proven BKPyV-associated nephropathy was diagnosed by the local pathologist at each center according to the respective most recent Banff classification [11].

### Evaluation of graft function and histopathology 

Graft function was assessed as estimated glomerular filtration rate (eGFR) which was calculated according to the revised Schwartz formula: eGFR = (mL/min per 1.73 m^2^) = 0.413 × [height (cm)/serum creatinine (mg/dL)] [12, 13]. Graft dysfunction was defined as an eGFR < 30 mL/min·1.73 m^2^ or an eGFR decline ≥ 50% of baseline (at month 1 post-transplant). In all cases, ABMR was a histopathologic diagnosis, either by indication biopsy or by protocol biopsy. Biopsy-proven rejection episodes (T-cell mediated rejection (TCMR) or ABMR and other histopathologic findings) were diagnosed by the local pathologist at each center according to the respective most recent Banff Working Classification of kidney transplant pathology. All inconclusive histopathology reports were re-evaluated by an experienced nephropathologist (R.W.) and reassessed according to the Banff 2019 classification scheme [11] to ensure consistent evaluation of histopathology data. ABMR was classified as acute ABMR, chronic active ABMR, or chronic inactive ABMR. Cases of mixed TCMR/ABMR (14 biopsies in 13 patients) were classified according to the underlying ABMR category and included in the respective ABMR analysis (resulting in 13 additional cases with acute ABMR). Three patients (0.9%) developed chronic inactive ABMR after chronic active ABMR episodes, and thus, chronic inactive ABMR was not included in this analysis.

### Statistical methods

Results for continuous variables are presented as mean ± standard deviation (SD) or median and interquartile range (IQR) according to the distribution. Categorical parameters are expressed as the number and percentage of patients. The Shapiro–Wilk test was used to assess whether the data were normally distributed. Univariate analyses were performed by using Cox proportional hazards models (hazard ratios with 95% confidence intervals) and Kaplan–Meier analysis. Patients who were lost to follow-up or did not reach an observation period of 5 years post-transplant were censored as having no event. Because graft loss is a rare event in pediatric kidney transplant recipients, we used a composite endpoint called allograft dysfunction as the primary outcome measure, defined as either graft loss, or an eGFR ≤ 30 mL/min per 1.73 m^2^ or a ≥ 50% decline from baseline eGFR at month 1 post-transplant, whichever occurred first. In 30 patients, eGFR was greater than 120 mL/min/1.73 m^2^ at month 1 post-transplant and was set at 120 mL/min/1.73 m^2^. The association of baseline characteristics at the time of transplantation and time-dependent covariates (rejection episodes, HLA-DSA) with the development of ABMR or with graft dysfunction was determined by multivariable Cox regression analysis. All covariates were assessed primarily in the univariate analysis and factors with a *p* value ≤ 0.2 were considered for inclusion in the multivariable models. Nonsignificant covariates were progressively eliminated using a stepwise selection method (*p* < 0.05). Multivariable models were adjusted for center effects, and all models and parameters were tested for violation of the proportional hazards assumption by plotting log-minus-log survival curves and by examining the scaled Schoenfeld residuals and the continuous residuals for the log-linearity assumption. Graft survival probabilities for acute ABMR or chronic active ABMR were estimated using a semi-Markov multistate model approach and a “clock reset” model: time was reset to zero each time the patient entered a new state (KTx, acute ABMR, or chronic active ABMR). Reported *p* values were two-tailed and were considered statistically significant if less than 0.05. All analyses were exploratory and not confirmatory. Statistical analysis was performed using the R (v4.0.2) computing environment with the CRAN “survival” and”coxme” packages https://cran.rproject.org/web/packages/survival/index.html.

## Results

We enrolled 337 eligible pediatric kidney transplant recipients from 21 European centers in this study. Patients who had undergone more than one kidney transplantation were only evaluated for the most recent transplantation. Patient demographics, clinical parameters, and initial immunosuppressive regimen are shown in Table 1, and the type of primary kidney disease is shown in Supplementary Table 1. The median age at transplantation was 10.9 years (IQR, 5.3–14.9). A total of 59.9% of patients were male and 30.3% had received a living donor kidney transplant. Forty-one patients (12.2%) had received a previous transplant and 25 patients (7.4%) had pre-formed HLA-DSA. The mean number of HLA mismatches was 2.88 (SD ± 1.31). Most patients (79.8%) received a tacrolimus-based immunosuppressive regimen. One hundred seventy-five patients (51.9%) received induction therapy, 43.0% an IL-2 receptor antibody, 7.4% thymoglobulin, and 1.5% rituximab (Table 1). This was an immunologically low-risk cohort: only 3.9% of patients had been desensitized for pretransplant donor-specific HLA-DSA. No ABO blood group incompatible kidney transplantation had been performed. One-year patient and graft survival rates were 100%; at 5 years post-transplant, patient and graft survival rates (uncensored for death) were 98.7% and 95.6% (Kaplan–Meier analysis), respectively. The mean follow-up was 4.01 ± 1.42 years.

**Table 1:** Clinical features at the time of transplantation associated with the development of antibody-mediated rejection in univariable Cox proportional hazard regression analyses

Parameter	Entire cohort (*n* = 337)	Without ABMR (*n* = 295)	ABMR^#^ (*n* = 42)	HR	*p* value
Age at KTx, years (median, IQR)	10.9 (5.3–14.9)	10.7 (4.9–14.7)	12.5 (7.9–16.2)	1.06 (1.01–1.13)	0.027
10–15 years^1^	99 (29.4)	87 (29.5)	12 (28.6)	1.39 (0.65–2.97)	0.395
> 15 years	80 (23.7)	66 (22.4)	14 (33.3)	2.43 (1.16–5.07)	0.018
Male sex, *n* (%)	202 (59.9)	174 (59.0)	28 (66.7)	1.58 (0.82–3.07)	0.172
> 1 KTx, *n* (%)	41 (12.2)	32 (10.8)	9 (21.4)	2.39 (1.14–5.02)	0.022
Living donation, *n* (%)	102 (30.3)	88 (29.8)	14 (33.3)	1.16 (0.60–2.22)	0.660
Preemptive donation, *n* (%)	83 (24.6)	70 (23.7)	13 (31.0)	1.32 (0.68–2.56)	0.415
Donor age, years (median, IQR)	35.0 (14.0–44.0)	33.0 (13.0–44.0)	40.0 (17.3–46.8)	1.02 (1.00–1.04)	0.102
Cold ischemia time, hours (median, IQR)	10.0 (3.1–14.8)	10.2 (3.1–14.8)	9.51 (3.3–14.6)	0.98 (0.94–1.03)	0.393
HLA mismatch, *n* (mean ± SD)	2.88 ± 1.31	2.83 ± 1.29	3.24 ± 1.38	1.25 (0.99–1.57)	0.057
HLA-DR mismatch, *n* (mean ± SD)	0.92 ± 0.66	0.88 ± 0.66	1.14 ± 0.65	1.80 (1.13–2.90)	0.014
Delayed graft function, *n* (%)	26 (7.7)	21 (7.1)	5 (11.9)	1.73 (0.68–4.43)	0.249
Preformed HLA-DSA, *n* (%)	25 (7.4)	18 (6.1)	7 (16.7)	2.37 (1.02–5.53)	0.045
CMV IgG negative recipient and donor, *n* (%)	115 (34.1)	108 (36.6)	7 (16.7)	0.39 (0.17–0.88)	0.024
EBV IgG negative recipient and donor, *n* (%)	49 (14.5)	42 (14.2)	7 (16.7)	1.21 (0.54–2.75)	0.643
Initial immunosuppressive regimen^2^					
Desensitization procedure, *n* (%)	13 (3.9)	12 (4.1)	1 (2.4)	0.62 (0.09–4.58)	0.648
IL-2R antibody induction, *n* (%)	145 (43.0)	128 (43.4)	17 (40.5)	0.99 (0.53–1.85)	0.981
Thymoglobulin, *n* (%)	25 (7.4)	19 (6.4)	6 (14.3)	2.83 (1.18–6.76)	0.019
Rituximab, *n* (%)	5 (1.5)	5 (1.7)	0		NA
Tacrolimus, *n* (%)	269 (79.8)	236 (80.0)	33 (78.6)	0.84 (0.40–1.77)	0.647
Ciclosporin, *n* (%)	68 (20.2))	59 (20.0)	9 (21.4)	1.18 (0.57–2.50)	0.647
Mycophenolate mofetil, *n* (%)	293 (86.9)	258 (87.5)	35 (83.3)	0.92 (0.39–2.20)	0.857
Everolimus, *n* (%)	16 (4.8)	16 (5.4)	0		NA
Azathioprine, *n* (%)	28 (8.3)	21 (7.1)	7 (16.7)	1.77 (0.74–4.25)	0.201
Glucocorticoids, *n* (%)	328 (97.3)	287 (97.3)	41 (97.6)	0.87 (0.15–5.06)	0.878

*ABMR* antibody-mediated rejection, *DSA* donor-specific antibodies, *HLA* human leukocyte antigen, *HR* hazard ratio, *IL-2R* interleukin 2 receptor, *KTx* kidney transplantation

^1^Age at KTx < 10 years is the reference group (HR = 1)

^2^Until day 30 post-transplant

^#^ABMR group includes acute ABMR, chronic active ABMR, and mixed rejections

**Fig. 1:**
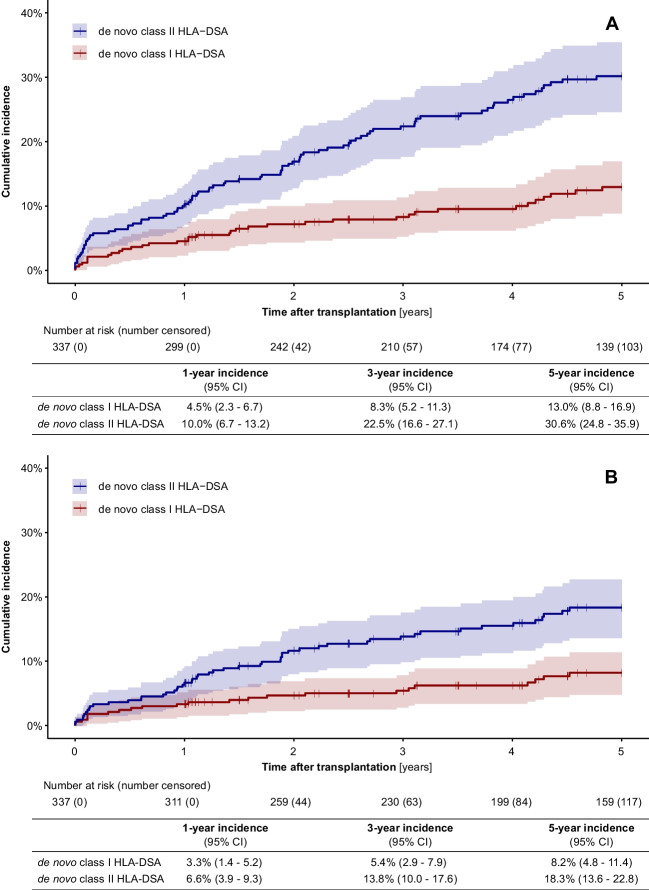
Cumulative incidence of de novo HLA-DSA during the first 5 years post-transplant. The red line indicates class I HLA-DSA, the blue line indicates class II HLA-DSA, and the shaded areas indicate the respective 95% confidence intervals. **A** Data reported by the respective transplant centers according to the center-specific cut-off for HLA-DSA positivity. **B** Data calculated using a uniform MFI cut-off value of 1400

Two thousand three hundred and ninety-two HLA-DSA measurements were reported. Ninety-four sera (3.9%) were classified as class I HLA-DSA positive only, 451 sera (18.9%) as class II HLA-DSA positive only, and 92 sera (3.8%) as both class I and class II HLA-DSA positive. Twenty-five of 337 recipients (7.4%) had known HLA-DSA at the time of transplantation; of these, 8 (32%) had only class I, 12 (48%) had only class II, and 5 (20%) had both class I and class II HLA-DSA. The median number of HLA-DSA measurements per patient was 6.0 (IQR, 3.0–10.0). Figure 1 shows the cumulative incidence of de novo HLA-DSA stratified by HLA class over the 5-year post-transplant observation period.

The data in Fig. 1A and B correspond to the data reported by the respective transplant centers according to the center-specific cut-off for HLA-DSA positivity. Throughout the 5-year observation period, the incidence of de novo class II HLA-DSA was approximately twice that of de novo class I HLA-DSA. The cumulative incidence of de novo class I HLA-DSA was 4.5% in year 1, 8.3% in year 3, and 13% in year 5; the corresponding data for de novo class II HLA-DSA were 10%, 22.5%, and 30.6%, respectively. The median MFI of the immunodominant de novo class I HLA-DSA at the time of first detection was 1928 (IQR, 1000–2607) and that of de novo class II HLA-DSA was 1994 (IQR, 913–6779). The median of the peak MFI of the immunodominant class I HLA-DSA per patient was 2000 (IQR, 1042–3407) and 3727 (IQR, 1278–11,568) for class II HLA-DSA. The data for persistent de novo HLA-DSA, defined as de novo HLA-DSA with at least two consecutive positive measurements [14], are shown in Supplementary Fig. 1a. In terms of immunosuppressive strategies, a greater proportion of patients with de novo HLA-DSA in the first year post-transplant received a ciclosporin-based initial immunosuppressive regimen compared to de novo HLA-DSA negative patients (Supplementary Table 2).

De novo HLA-DSA data using a uniform post-transplant MFI cut-off value of 1400, as suggested for multicenter studies [9], are presented in Fig. 1B. The respective incidences were lower than those with the center-specific cut-off at all observation points. The cumulative incidences of persistent de novo HLA-DSA, using a uniform MFI cut-off value of 1400, are shown in Supplementary Fig. 1b.

Six hundred fifty-four kidney transplant biopsies were reported in 337 patients, 397 indication biopsies, and 257 protocol biopsies including preimplant and post-perfusion biopsies (*n* = 26). The most frequent time points for surveillance biopsies were 6 months (*n* = 78) and 12 months post-transplant (*n* = 74). In indication biopsies, the most frequently reported diagnosis was borderline rejection (29.7%), followed by interstitial fibrosis and tubular atrophy (IFTA) (19.4%) and T-cell mediated rejection (8.8%). As expected, the corresponding pathologies detected in surveillance biopsies were lower, with borderline rejection (9.7%) and T cell-mediated rejection (5.1%) being the most common histopathologic diagnoses (Table 2).

**Table 2:** Graft histopathology according to the respective most recent Banff Working Classification of kidney transplant pathology

Entity	Overall (*n* = 654)	Indication biopsies (*n* = 397)	Surveillance biopsies (*n* = 257)
Acute ABMR, *n* (%)	25 (3.82)	23 (5.79)	2 (0.78)
Chronic active ABMR, *n* (%)	23 (3.52)	20 (5.04)	3 (1.17)
Mixed ABMR/TCMR, *n* (%)	14 (2.14)	13 (3.27)	1 (0.39)
Borderline, *n* (%)	143 (21.9)	118 (29.7)	25 (9.73)
TCMR (Banff I-III), *n* (%)	48 (7.34)	35 (8.82)	13 (5.06)
Chronic TCMR, *n* (%)	9 (1.38)	8 (2.02)	1 (0.39)
BKPyV nephropathy, *n* (%)	31 (4.74)	26 (6.55)	5 (1.95)
IFTA (grade II/III), *n* (%)	83 (12.7)	77 (19.4)	6 (2.33)

*ABMR* antibody-mediated rejection, *TCMR* T-cell mediated rejection, *BKPyV* BK polyomavirus, *IFTA* interstitial fibrosis and tubular atrophy

**Fig. 2:**
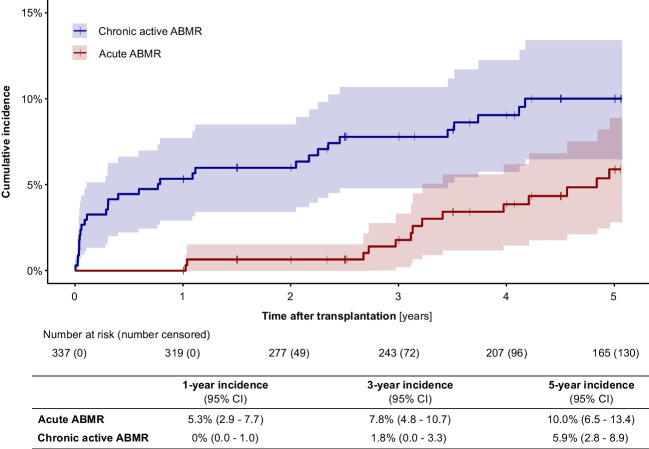
Cumulative incidence of acute antibody-mediated rejection (ABMR) and chronic active ABMR during the first 5 years post-transplant. The red line indicates acute ABMR; the blue line indicates chronic active ABMR; shaded areas indicate 95% confidence interval

Forty-two of 337 patients (12.5%) developed ABMR during the observation period. Nineteen of these 42 patients (45.2%) had acute ABMR, 16 of 42 patients (38.1%) had chronic active ABMR, and 13 of 42 patients (31.0%) had mixed cellular and ABMR (a patient could have more than one ABMR episode). Figure 2 shows the cumulative incidence of acute ABMR and chronic active ABMR during the 5 years post-transplant. During the first 3 years, acute ABMR occurred more frequently than chronic ABMR; thereafter, the incidences slowly converged. At 5 years post-transplant, the cumulative incidence of acute ABMR was 10% and that of chronic active ABMR 5.9%. In addition, the incidence rates of de novo HLA-DSA and ABMR in the subgroup of low-risk patients with a first transplant and with no preformed HLA-DSA are shown in Supplementary Figs. 2 and 3.

Table 1 shows baseline demographic and transplant characteristics associated with the development of ABMR by univariate Cox regression analysis. Older age, re-transplantation, a higher number of HLA-DR mismatches, preformed HLA-DSA, and a CMV serostatus other than “cytomegalovirus (CMV) IgG negative recipient (R-) and CMV IgG negative donor (D-)” were associated with the occurrence of ABMR. The proportion of patients with CMV D-/R- status did not differ significantly between different age groups (*p* = 0.15). A negative CMV-IgG donor status was associated with a reduced risk for ABMR development, although this did not reach statistical significance (HR 0.53 (IQR 0.28–1.02), *p* = 0.069). Furthermore, there was no association between CMV D-/R- status and de novo HLA-DSA development (*p* = 0.788). The initial immunosuppressive therapy did not differ between the groups (Table 3). The EBV serostatus was not associated with the occurrence of ABMR. Induction therapy with IL-2R antibodies was not associated with the development of de novo HLA-DSA or ABMR, even when the analysis was corrected for center effects in a multivariable model (de novo HLA-DSA *p* = 0.483, ABMR *p* = 0.220). A total of 24 out of the 25 patients (96%) who received induction therapy with thymoglobulin underwent retransplantation. Therefore, in contrast to the univariate results shown in Table 1, the analysis adjusting for center effects and retransplantation showed no significant association between thymoglobulin induction and the occurrence of ABMR (*p* = 0.437). A similar result was observed for de novo HLA-DSA development (*p* = 0.202). Table 4 shows the risk factors for the development of ABMR by multivariable Cox regression analysis with time-varying covariables (HLA-DSA). Higher HLA-DR mismatch was a significant risk factor for ABMR, whereas CMV R-/D- was associated with a lower risk. As expected, the presence of de novo class II HLA-DSA only was a significant risk factor for ABMR. This risk was further increased in patients who were double positive for both HLA class I and class II HLA-DSA. Patients who developed only class I HLA-DSA did not have an increased risk of ABMR. Furthermore, the risk of developing ABMR was elevated in patients with higher HLA-DSA levels or MFI-load, as indicated by the sum of all HLA-DSA MFI values at the time of the initial HLA-DSA positive measurement (Supplementary Table 3).

**Table 3:** Immunosuppressive regimens in patients with the CMV serostatus “donor and recipient seronegative” and in patients with the CMV serostatus “donor and/or recipient seropositive”

Parameter				
	Entire cohort (*n* = 337)	CMV serostatus donor negative/recipient negative (*n* = 115)	CMV serostatus donor and/or recipient positive (*n* = 222)	*p value*
Initial immunosuppressive regimen^1^				
Desensitization procedure, *n* (%)	13 (3.86)	2 (1.74)	11 (4.95)	0.232^#^
IL-2R antibody induction, *n* (%)	145 (43.0)	42 (36.5)	103 (46.4)	0.104
Thymoglobulin, *n* (%)	25 (7.42)	6 (5.22)	19 (8.56)	0.381^#^
Tacrolimus, *n* (%)	269 (79.8)	88 (76.5)	181 (81.5)	0.346
Mycophenolate mofetil, *n* (%)	293 (86.9)	102 (88.7)	191 (86.0)	0.606
Azathioprine, *n* (%)	28 (8.31)	6 (5.22)	22 (9.91)	0.152^#^
Glucocorticoids, *n* (%)	328 (97.3)	111 (96.5)	217 (97.7)	0.497
Maintenance immunosuppressive regimen^2^				
Tacrolimus, *n* (%)	267 (79.2)	87 (75.7)	180 (81.1)	0.269
Mycophenolate mofetil, *n* (%)	256 (76.0)	89 (77.4)	167 (75.2)	0.812
Azathioprine, *n* (%)	28 (8.31)	7 (6.09)	21 (9.46)	0.405^#^
Glucocorticoids, *n* (%)	283 (84.0)	96 (83.5)	187 (84.2)	0.909

*IL-2R* interleukin 2 receptor

^1^Until day 30 posttransplant

^2^At 12 months posttransplant

The respective frequencies of the immunosuppressive regimens between the two patient cohorts were compared by Chi-square test or Fisher’s exact test (marked by ^#^)

**Table 4:** Risk factors for the development of antibody-mediated rejection

Risk factors for ABMR^#^ development	Unadjusted HR (95% CI)	*p* value	Adjusted HR (95% CI)	*p* value
Age at KTx, years	1.06 (1.01–1.13)	0.027	1.06 (1.00–1.13)	0.048
> 1 KTx (> 1 versus 1)	2.39 (1.14–5.02)	0.022	1.85 (0.85–4.04)	0.122
HLA-DR mismatch (0 versus 1 or 2)	1.80 (1.13–2.90)	0.014	1.85 (1.13–3.02)	0.015
CMV IgG negative recipient and negative donor	0.39 (0.17–0.88)	0.024	0.40 (0.18–0.90)	0.027
Preformed HLA-DSA	2.37 (1.02–5.53)	0.045	1.30 (0.54–3.14)	0.563
TCMR/borderline rejection	1.14 (0.53–2.43)	0.743		
BKPyV nephropathy	0.99 (0.13–7.31)	0.99		
de novo class I HLA-DSA only^1,2^	3.07 (0.73–13.0)	0.127		
de novo class II HLA-DSA only^1,2^	3.38 (1.49–7.63)	0.003	3.78 (1.62–8.82)	0.002
de novo class I and II HLA-DSA^1,2^	4.42 (1.68–11.6)	0.003	5.00 (1.75–14.2)	0.001

*ABMR* antibody-mediated rejection, *CI* confidence interval, *DSA* donor-specific antibodies, *HLA* human leukocyte antigen, *HR* hazard ratio, *KTx* kidney transplantation, *TCMR* T-cell mediated rejection

^1^De novo HLA-DSA and histopathologic diagnoses were regarded as time-dependent co-variables

^2^Data were reported by the respective transplant centers according to the center-specific cut-off for HLA-DSA positivity

^#^ABMR includes acute ABMR, chronic active ABMR, and mixed rejections

Table 5 shows the different procedures and agents used by the centers, either alone or in combination. Episodes of acute ABMR (*n* = 19) were mostly treated with steroid pulse therapy (17/19, 89.5%) in combination with rituximab (13/19, 68.4%), either plasmapheresis or immunoadsorption (10/19, 52.6%), and intravenous immunoglobulin G (IVIG) (10/19, 42.1%); other agents (eculizumab, thymoglobulin, bortezomib) were used less frequently. Chronic active ABMR (*n* = 18) was mostly treated with optimization of maintenance immunosuppressive therapy (17/18, 94.4%), IVIG (10/18, 55.6%), and rituximab (7/18, 38.9%); other agents (thymoglobulin, bortezomib, tocilizumab) were used less frequently. Due to the relatively small number of ABMR episodes and the heterogeneity of therapeutic interventions, their respective efficacy in terms of graft function preservation could not be analyzed.

**Table 5:** Treatment strategies for ABMR episodes (*n* = 51)

Treatment strategies		*N* (%)
Acute ABMR		*n* = 19
	Rituximab, IVIG, optimization, and/or steroid pulse	5 (26.3%)
	Rituximab, PF/IA, steroid pulse, w/o IVIG	4 (21.1%)
	Eculizumab or rituximab, thymoglobulin, PF/IA, steroid pulse	3 (15.8%)
	Thymoglobulin, steroid pulse, PF/IA, or optimization	2 (10.5%)
	Steroid pulse, w/o IVIG	2 (10.5%)
	Optimization	2 (10.5%)
	Bortezomib, rituximab, PF/IA, optimization, steroid pulse, IVIG	1 (5.3%)
Chronic active ABMR		*n* = 18
	Rituximab, IVIG, optimization, and/or steroid pulse	5 (27.8%)
	Optimization or no treatment	4 (22.2%)
	Thymoglobulin, optimization, w/o steroid pulse	2 (11.1%)
	Rituximab, PF/IA, optimization, IVIG, w/o steroid pulse	2 (11.1%)
	IVIG, steroid pulse, optimization	2 (11.1%)
	Bortezomib, steroid pulse, optimization	1 (5.6%)
	Tocilizumab, PF/IA, IVIG, optimization	1 (5.6%)
	Thymoglobulin, PF/IA, steroid pulse	1 (5.6%)
Mixed ABMR/TCMR		*n* = 14
	Rituximab, PF/IA, steroid pulse, IVIG, and/or optimization	5 (35.7%)
	Rituximab, steroid pulse, w/o IVIG, w/o optimization	3 (21.4%)
	PF/IA, steroid pulse, w/o IVIG and optimization	2 (14.3%)
	Eculizumab or thymoglobulin, rituximab, PF/IA, steroid pulse, IVIG, w/o optimization	2 (14.3%)
	Steroid pulse, w/o IVIG and optimization	2 (14.3%)

*ABMR* antibody-mediated rejection, *IVIG* intravenous immunoglobulin G, *PF* plasmapheresis, *IA* immunoadsorption, *Optimization* increase or change of maintenance immunosuppression, *w/o* with or without

**Fig. 3:**
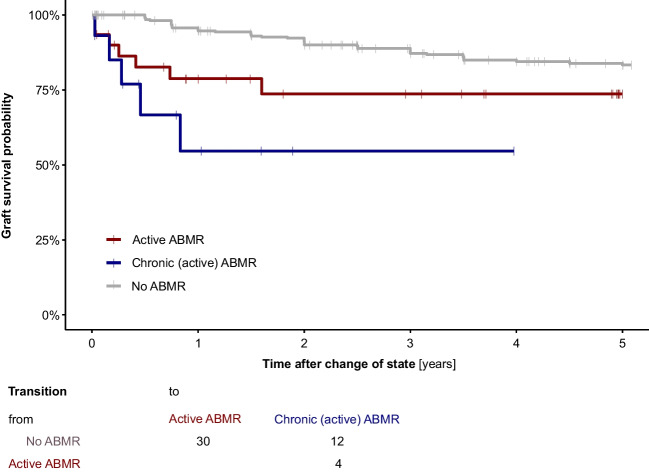
Allograft survival probability of acute ABMR (*n* = 30, red line) and chronic active ABMR (*n* = 16, blue line). Patients with mixed ABMR and TCMR (*n* = 13) were assigned to the ABMR category determined by the histologic lesions, in this analysis always the acute ABMR category. Analysis was performed using a semi-Markov multi-state model. Follow-up time is taken from the time of the event (clock reset model). The grey line indicates patients without ABMR

The graft survival probability for patients with chronic active ABMR was remarkably lower than for patients with acute ABMR, as analyzed by the semi-Markov multistate model (clock reset model) (Fig. 3). These data show that ABMR remains a difficult complication to treat, with nearly 25% of patients having lost their graft within 1 year of diagnosis of acute ABMR, and nearly 50% of patients lost their graft within 1 year of diagnosis of chronic ABMR.

During the observation period of up to 5 years, 55 patients (16.3%) reached the composite endpoint of graft dysfunction: *n* = 10 graft loss, *n* = 20 eGFR < 30 mL/min per 1.73 m^2^, and *n* = 25 > 50% decline from baseline eGFR at month 1 post-transplant. Table 6 shows the risk factor analysis for the composite endpoint graft dysfunction. In the multivariable Cox regression analysis with time-dependent covariates, the presence of BKPyV nephropathy (*n* = 19 patients) was associated with the highest risk of graft dysfunction, followed by ABMR (*n* = 42 patients), TCMR (*n* = 117 patients), and older donor age (increased risk of 0.02 per year older donor age).

**Table 6:** Risk factor analysis for graft dysfunction (defined as graft loss, an eGFR < 30 mL/min·1.73 m^2^ or an eGFR decline ≥ 50% of baseline)

Risk factors for graft dysfunction	Unadjusted HR (95% CI)	*p* value	Adjusted HR (95% CI)	*p* value
Age at KTx, years	0.97 (0.93–1.02)	0.266		
Male sex	1.14 (0.66–1.96)	0.640		
> 1 KTx	1.81 (0.91–3.58)	0.092		
Living donation	1.44 (0.84–2.48)	0.189		
Donor age, years^2^	1.02 (1.01–1.04)	0.005	1.02 (1.00–1.04)	0.007
Cold ischemia time	0.98 (0.94–1.02)	0.289		
HLA-DR mismatch (0 versus 1 or 2)	1.09 (0.73–1.62)	0.685		
Delayed graft function	1.19 (0.48–2.99)	0.710		
Time from dialysis to KTx (> 21 months)	1.23 (0.72–2.09)	0.447		
IL-2R antibody induction	0.67 (0.38–1.17)	0.161		
Potential recurrent disease^1^	1.14 (0.51–2.51)	0.752		
BKPyV nephropathy^2,3^	5.45 (2.46–12.1)	< 0.001	7.20 (3.15–16.5)	< 0.001
ABMR^2,3^	4.06 (2.13–7.73)	< 0.001	3.30 (1.72–6.33)	< 0.001
TCMR and treated borderline rejection^2,3^	2.59 (1.52–4.42)	0.001	2.48 (1.44–4.30)	0.001

*ABMR* antibody-mediated rejection, *BKPyV* BK polyomavirus, *CI* confidence interval, *HLA* human leukocyte antigen, *HR* hazard ratio, *IL-2R* interleukin 2 receptor, *KTx* kidney transplantation, *TCMR* T cell-mediated rejection

^1^Potentional recurrent disease included: atypical hemolytic uremic syndrome, non-genetic focal segmental glomerulosclerosis, type I and type II membranoproliferative glomerulonephritis, IgA nephropathy, membranous glomerulonephritis, primary hyperoxaluria, and ANCA-associated vasculitis

^2^Multivariable Cox regression model with time-variable covariables adjusted for center effect

^3^Histopathologic diagnoses were treated as time-dependent variables

## Discussion

To our knowledge, this is the first multicenter study in pediatric kidney transplant recipients which provides detailed information regarding the incidence, risk factors, management strategies, graft survival probability of ABMR, and incidence of de novo HLA-DSA. We observed that the cumulative incidences of de novo class II HLA-DSA at 1, 3, and 5 years post-transplant were 10%, 22.5%, and 30.6%, respectively; the cumulative incidence of de novo class I HLA-DSA was approximately half that. These incidence rates are comparable to studies from Ginevri et al. and Cioni et al. who reported de novo HLA-DSA incidence rates between 30 and 34% at 5 years post-transplant [15, 16]. Miettinen et al. reported in a cross-sectional study at 3 to 5 years post-transplant a prevalence of 20% of de novo HLA-DSA, while Ettenger et al. reported a slightly higher incidence rate early post-transplant (19.8% at 1 year post-transplant), similar to that reported by Chaudhuri et al. (22% at 2 years post-transplant) [4, 17, 18]. Possible explanations for these differences include the baseline immunologic risk of the study participants, the definition of HLA-DSA positivity [19], the frequency of DSA testing, the type of induction and maintenance immunosuppressive therapy, and different adjustment strategies of immunosuppressive therapy in response to the occurrence of de novo HLA-DSA. Indeed, when we used a uniform MFI cut-off value of 1400 to define HLA-DSA positivity, as suggested for multicenter studies [9], the calculated respective incidence rates of class I and class II HLA-DSA at all time points of observation were somewhat lower (Fig. 1). This observation underscores the urgent need for harmonization of HLA-DSA reporting and definition of HLA-DSA positivity, as outlined in the recent STAR 2022 Working Group report [20]. Furthermore, the study population is a European collective in which only about half of the patients received induction therapy due to uncertainty regarding the therapeutic benefit in immunologically low- and standard-risk cases [21, 22].

 We observed a cumulative incidence of acute ABMR of 10% and of chronic active ABMR of 5.9% at 5 years post-transplant. Previous single-center studies with lower patient numbers found lower ABMR rates ranging from 5 to 7.7% [23, 24]. One study reported a diagnosis of ABMR in 21 of 114 (18.4%) pediatric kidney transplant recipients at a median follow-up of 4.8 years [16], while Steggerda et al. reported acute ABMR in 11 of 118 (9.3%) patients and chronic active ABMR in 5 of 118 (4.2%) with a median time to diagnosis of ABMR of 3.7 years in the subgroup with de novo HLA-DSA [25]. In adult kidney transplant recipients, a recent systematic review reported an incidence rate of 1.1 to 21.5% for acute ABMR and of 7.5 to 20.1% for chronic ABMR over a follow-up period of up to 10 years [2]. This variability may be attributed to differences in the baseline immunological risk of study participants and the evolving definitions of ABMR, which have undergone significant changes over the past two decades with the advent of the Banff histopathologic grading scheme.

In our study, independent risk factors for the development of ABMR by multivariate Cox regression analysis were HLA-DR mismatch and the presence of de novo HLA-DSA, especially the concurrent positivity for class I and class II HLA-DSA, whereas the CMV serostatus R-/D- was associated with a lower risk. While the first two risk factors have been previously described in both pediatric [3, 15, 19] and adult [1, 2] kidney transplant recipients, the observation that the CMV serostatus R-/D- is associated with a lower risk of ABMR in pediatric kidney transplant recipients is novel and interesting. Possible explanations for a causal relationship between CMV infection and ABMR include the following: CMV induces the expression of B-cell activating factor (BAFF), a cytokine involved in B-cell homeostasis that communicates survival and growth signals to B cells and virus-specific plasma cells via the BAFF receptor, TACI (the calcium modulator, the cyclophilin ligand interactor), and B cell maturation antigen (BCMA) receptors. These molecules of the BAFF system have also been suggested as biomarkers for the development of alloantibodies and graft dysfunction [26]. Recently, early CMV reactivation in kidney transplant recipients has been shown to be associated with high levels of BCMA transcript expression prior to transplantation [27]. Furthermore, in vitro studies have demonstrated that NK cells from CMV-positive patients exhibit heightened overall NK cell reactivity, which enhances antibody-dependent reactivity, including reactivity to anti-HLA-specific antibodies. This may potentially increase the risk of developing ABMR [28]. Others have discussed the potential role of CD4 + T cells of CMV-seropositive patients, which produce interferon-γ and induce both MHC class II and adhesion molecule overexpression on endothelial cells. Alternatively, a direct role of CMV-induced CD16 + γδ T cells in ABMR has been proposed [29]. Furthermore, there is evidence to suggest that subclinical CMV infection may be associated with chronic allograft injury [30]. Saldan et al. reported an association between early CMV DNAemia and ABMR in heart-transplanted patients [31]. However, Boutolleau et al. did not find a similar association [32].

In our study, further analyses pertaining to the impact of cytomegalovirus (CMV) and Epstein-Barr virus (EBV) infections or DNAemia could not be conducted due to the limited availability of comprehensive data. Nevertheless, the precise mechanisms by which CMV may contribute to the development of ABMR remain incompletely understood and require further investigation.

Using a multivariable Cox regression model with time-varying covariates adjusted for center effect, we found that immunologic risk factors for graft dysfunction were ABMR and TCMR. While several treatment options are available for the treatment of TCMR, such as steroid pulse therapy, anti-thymocyte globulin, and optimization of immunosuppressive therapy with calcineurin inhibitors, there are no agents approved by the Food and Drug Administration (FDA) in the USA for the treatment of ABMR. In particular, chronic active ABMR has an unfavorable prognosis, as demonstrated by our analysis using the semi-Markov multistate model (Fig. 3). These data on the incidence of ABMR may be informative for future trial design in the pediatric population. Several investigational drugs are currently being tested for efficacy and safety in the treatment of chronic active ABMR, such as IL-6 antibody (clazakizumab [33] and tocilizumab [34]), or anti-complement C1s monoclonal antibody BIVV020 [35].

A 2019 Transplantation Society expert consensus [36] notes that studies on the treatment of ABMR each describe a different mix of interventions, further complicating the interpretation of results. Plasma exchange and IVIG, complement inhibitors, rituximab, imlifidase, ATG, splenectomy, bortezomib, cyclophosphamide, and interleukin-6 inhibitors are discussed. The consensus paper, while acknowledging the weak data, identifies the combination of plasma exchange and IVIG as the standard of care, but recommends that additional therapeutic interventions be considered to prevent organ loss (< 30 days post-transplant) or slow GFR decline (> 30 days). The potential benefits must be weighed against the consequences of increased immunosuppression. The absence of evidence for specific therapeutic strategies in ABMR is reflected in the heterogeneity of the reported regimens in our study. The data does not permit the identification of superior regimens, and therefore, no meaningful analysis can be performed or definitive conclusions drawn regarding success rates. This quandary is also reflected by a recently published analysis, which surveyed 52 transplant nephrologists from 18 European countries (46 from high-volume academic centers) about their practices in the treatment of chronic active ABMR [37]. The use of IVIG was 71%, apheresis 62%, rituximab 50%, tocilizumab 31%, bortezomib 6%, and eculizumab 4%.

In our study, a strong non-immunologic risk factor for graft dysfunction was the presence of BKPyV nephropathy. This risk factor was not analyzed in previous single-center studies in pediatric kidney transplant recipients on the outcome of ABMR [16] and may therefore have been overlooked. Our findings highlight the urgent need for effective antiviral treatment modalities for BKPyV nephropathy. Current treatment recommendations are to taper immunosuppressive therapy, which often secondarily increases the occurrence of adverse immunologic events such as the development of de novo HLA-DSA and graft rejection [38].

The strengths of our study are the multicenter design, the largest number of patients with ABMR analyzed to date, and the review of inconclusive biopsy reports by a single histopathologist using the most recent Banff Working Classification of kidney transplant pathology. Our study has several limitations, the most important of which is the retrospective study design, which is common to registry analyses in general. Another limitation is the lack of centralized laboratory assessment of HLA-DSA and the limited reporting of DQ-mismatches. The registry also does not collect reliable data on immunosuppressive drug adherence, a major risk factor for de novo HLA-DSA and rejection in adolescent and young adult kidney transplant recipients. We also did not systematically collect data on non-HLA antibodies, which may play a pathogenic role in a subset of patients with ABMR [39–42]. Also, quantitative data on proteinuria and detailed data on arterial hypertension, which are important risk factors for kidney transplant outcome [43], were not reported by many centers. However, registries reflecting the real-world scenario with heterogeneous immunosuppressive regimens and treatment protocols are potentially suitable to provide a broader view of outcomes and complications, especially in small cohorts such as pediatric kidney allograft recipients.

In conclusion, this multicenter registry study provides an estimate of the incidence of de novo HLA-DSA and ABMR in a large cohort of pediatric kidney transplant recipients. Our data confirm previous risk factors for the development of ABMR such as HLA-DR mismatch and the presence of de novo HLA-DSA. We add to the current literature the novel observation that the CMV serostatus D-/R- is associated with a lower risk of ABMR. Our data also highlight the importance of BKPyV nephropathy as a strong risk factor for allograft dysfunction. These data may enhance our understanding of the complex interplay of factors that contribute to the progressive decline of allograft function and may inform the design of future therapeutic trials to improve the outcome of kidney transplantation in this vulnerable patient population.

## Supplementary Information

Below is the link to the electronic supplementary material.

Graphical abstract (PPTX 125 KB)

Supplementary file2 (PDF 317 KB)

## Data Availability

The data that support the findings of this study are available from the corresponding author upon reasonable request.

## Abbreviations

ABMR: Antibody-mediated rejection
BKPyV: BK polyomavirus
CERTAIN: Cooperative European Paediatric Renal Transplant Initiative
CI: Confidence interval
CKD: Chronic kidney disease
CMV: Cytomegalovirus
DNA: Deoxyribonucleic acid
eGFR: Estimated glomerular filtration rate
HLA: Human leukocyte antigen
HLA-DSA: Donor-specific HLA antibodies
HR: Hazard ratio
IQR: Interquartile range
IVIG: Intravenous immunoglobulin
IL-2R: Interleukin 2 receptor
pKTR: Pediatric kidney transplant recipient
KTx: Kidney transplantation
MFI: Mean fluorescence intensity
SD: Standard deviation
TCMR: T-cell mediated rejection
