# Supplementary material for: Incidence, risk factors, management strategies, and outcomes of antibody-mediated rejection in pediatric kidney transplant recipients—a multicenter analysis of the Cooperative European Paediatric Renal Transplant Initiative (CERTAIN)
Source: Pediatr Nephrol. 2024 Sep 16;40(2):491–503. doi: 10.1007/s00467-024-06487-2 (PMC11666708; doi:10.1007/s00467-024-06487-2)
Supplement: Supplementary file 1 — Graphical abstract (PPTX 125 KB) [file 467_2024_6487_MOESM1_ESM.pptx]

## Slide 1
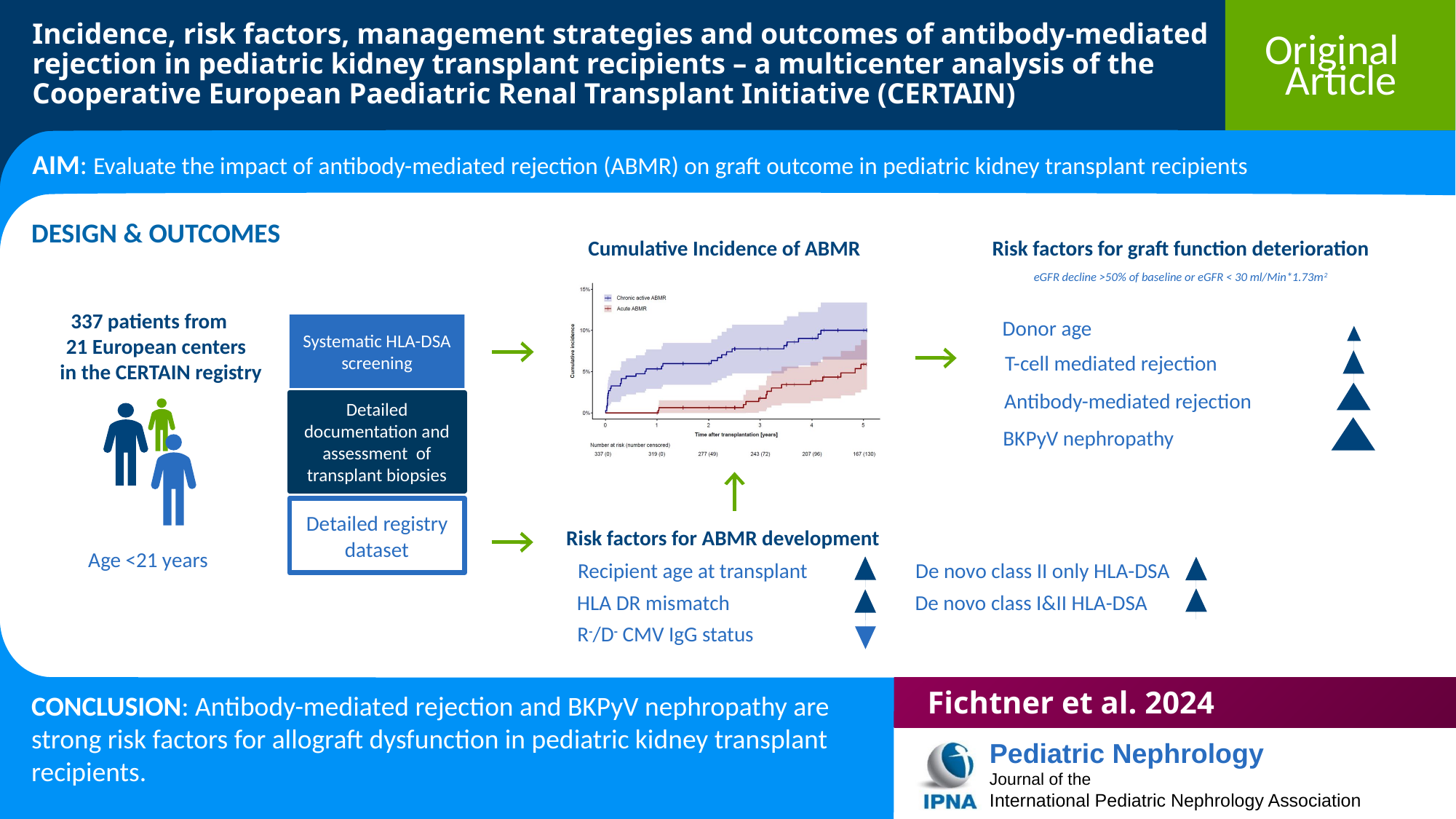

Incidence, risk factors, management strategies and outcomes of antibody-mediated rejection in pediatric kidney transplant recipients – a multicenter analysis of the Cooperative European Paediatric Renal Transplant Initiative (CERTAIN)
AIM: Evaluate the impact of antibody-mediated rejection (ABMR) on graft outcome in pediatric kidney transplant recipients
DESIGN & OUTCOMES
Risk factors for graft function deterioration
Cumulative Incidence of ABMR
eGFR decline >50% of baseline or eGFR < 30 ml/Min*1.73m2
337 patients from 21 European centers in the CERTAIN registry
Donor age
Systematic HLA-DSA screening
T-cell mediated rejection
Antibody-mediated rejection
Detailed documentation and assessment of transplant biopsies
BKPyV nephropathy
Detailed registry dataset
Risk factors for ABMR development
Age <21 years
De novo class II only HLA-DSA
Recipient age at transplant
HLA DR mismatch
De novo class I&II HLA-DSA
R-/D- CMV IgG status
Fichtner et al. 2024
CONCLUSION: Antibody-mediated rejection and BKPyV nephropathy are strong risk factors for allograft dysfunction in pediatric kidney transplant recipients.
