# Supplementary material for: Incidence, risk factors, management strategies, and outcomes of antibody-mediated rejection in pediatric kidney transplant recipients—a multicenter analysis of the Cooperative European Paediatric Renal Transplant Initiative (CERTAIN)
Source: Pediatr Nephrol. 2024 Sep 16;40(2):491–503. doi: 10.1007/s00467-024-06487-2 (PMC11666708; doi:10.1007/s00467-024-06487-2)
Supplement: Supplementary file 2 — Supplementary file2 (PDF 317 KB) [file 467_2024_6487_MOESM2_ESM.pdf]

Supplementary Information

Table S1. Primary kidney diseases

Parameter	Entire cohort (n = 337)	Without ABMR (n = 295)	ABMR (n = 42)	P value
Underlying kidney diseases				
CAKUT, n (%)	123 (36.5)	109 (36.9)	14 (33.3)	0.776
Cystic kidney disease, n (%)	59 (17.5)	52 (17.6)	7 (16.7)	0.990#
FSGS, n (%)	39 (11.6)	33 (11.2)	6 (14.3)	0.605#
Other, n (%)	29 (8.60)	23 (7.80)	6 (14.3)	0.232#
Syndrome-related kidney disease, n (%)	23 (6.82)	20 (6.78)	3 (7.14)	0.990#
aHUS or MPGN, n (%)	19 (5.64)	16 (5.42)	3 (7.14)	0.717#
Congenital nephrotic syndrome, n (%)	12 (3.56)	9 (3.05)	3 (7.14)	0.178#
IgA- or vasculitis-associated nephropathy, n (%)	6 (1.78)	6 (2.03)	0	1.00#
Unknown cause of kidney failure, n (%)	27 (8.01)	27 (9.15)	0	0.034#

CAKUT, congenital anomalies of the kidney and urinary tract. FSGS, focal-segmental glomerulosclerosis. aHUS, atypical hemolytic uremic syndrome. MPGN, membranoproliferative glomerulonephritis. The respective frequencies of the primary kidney diseses in the two patient cohorts were compared by Chi-square test or by Fisher's exact test (marked by #).

Table S2. Immunsuppressive regimens in patients with or without *de-novo* HLA-DSA (*dn*DSA) in the first year post-transplant

**Parameter**	***dn*DSA negative patients (n=303)**	***dn*DSA positive patients (n=34)**	**P value**
Initial immunosuppressive regimen[1]			
Desensitization procedure, n (%)	10 (3.30)	3 (8.82)	0.133[#]
IL-2R antibody induction, n (%)	133 (43.9)	12 (35.3)	0.698
Tacrolimus, n (%)	247 (81.5)	22 (64.7)	0.037
CsA, n (%)	56 (18.5)	12 (35.3)	0.037
Mycophenolate mofetil, n (%)	262 (86.5)	31 (91.2)	0.614
Azathioprine, n (%)	26 (8.58)	2 (5.88)	0.999[#]
Glucocorticoids, n (%)	294 (97.0)	34 (100)	0.607[#]
Maintenance immunosuppressive regimen[2]			
Tacrolimus, n (%)	243 (80.2)	25 (73.5)	0.373
Everolimus, n (%)	36 (11.9)	3 (8.82)	0.781[#]
Mycophenolate mofetil, n (%)	227 (74.9)	30 (88.2)	0.092
Azathioprine, n (%)	27 (8.91)	2 (5.88)	0.335[#]
Glucocorticoids, n (%)	252 (83.2)	32 (94.1)	0.134

IL-2R, interleukin 2 receptor. [1]Until day 30 posttransplant. [2]at 12 months posttransplant. The respective frequencies of the immunosuppressive regimens between the two patient cohorts were compared by Chi-square test or by Fisher's exact test (marked by [#]).

Table S3.

**Risk factors for ABMR development**	**Unadjusted HR (95% CI)**	***P* value**	**Adjusted HR (95% CI)**	***P* value**
Age at KTx, years	1.06 (1.01 - 1.13)	0.027	1.07 (1.01 - 1.14)	0.034
> 1 KTx (>1 versus 1)	2.39 (1.14 - 5.02)	0.022	1.56 (0.69 – 3.50)	0.283
HLA-DR mismatch	1.80 (1.13 - 2.90)	0.014	1.80 (1.09 - 2.96)	0.021
CMV IgG negative recipient and negative donor	0.39 (0.17 - 0.88)	0.024	0.44 (0.19 - 0.99)	0.044
Preformed HLA-DSA	2.37 (1.02 - 5.53)	0.045	1.54 (0.62 - 3.80)	0.348
TCMR / borderline rejection[1]	1.14 (0.53 - 2.43)	0.743		
BKPyV nephropathy[1]	0.99 (0.13- 7.31)	0.99		
de novo HLA-DSA sum MFI <5000[1,2]	3.66 (1.56 - 8.60)	0.003	2.93 (1.18 - 7.25)	0.020
de novo HLA-DSA sum MFI 5000 to 10000[1,2]	5.75 (1.31 - 25.2)	0.020	5.13 (1.17 - 22.5)	0.030
de novo HLA-DSA sum MFI > 10000[1,2]	11.9 (4.37 - 32.6)	< 0.001	7.74 (2.75 - 21.8)	< 0.001

ABMR, antibody-mediated rejection; CI, confidence interval; DSA, donor-specific antibodies; HLA, human leukocyte antigen; HR, hazard ratio; KTx, kidney transplantation; TCMR, T-cell mediated rejection. [1]*de novo* HLA-DSA and histopathologic diagnoses were regarded as time-dependent co-variables. [2]sum MFI, sum of all *de novo* HLA-DSA antibodies at time of first positive measurement.

Fig. S1. Cumulative incidence of peristent de *novo* HLA-DSA during the first 5 years posttransplant. **Panel a**, data reported by the respective transplant centers according to the center-specific cut-off for HLA-DSA positivity; **panel b**, data calculated using a uniform MFI cut-off value of 1400.

a

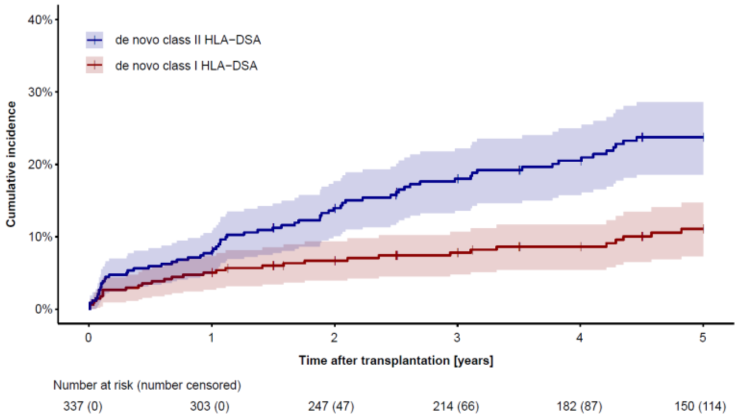

	1-year incidence (95% CI)	3-year incidence (95% CI)	5-year incidence (95% CI)
de novo class I HLA-DSA	4.5% (2.3 – 6.7)	7.8% (4.8 – 10.7)	11.1% (7.3 – 14.7)
de novo class II HLA-DSA	7.8% (5.1 – 10.9)	18.0% (13.5 – 22.1)	23.7% (18.5 – 28.5)

b

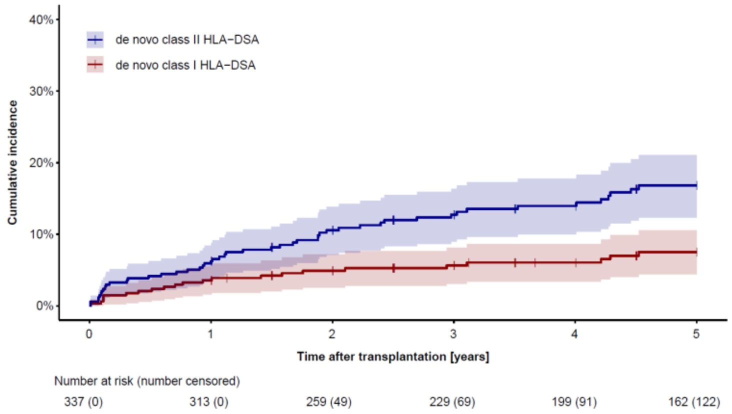

	1-year incidence (95% CI)	3-year incidence (95% CI)	5-year incidence (95% CI)
de novo class I HLA-DSA	3.0% (1.1 – 4.8)	5.0% (2.6 – 7.4)	6.9% (3.9 – 9.8)
de novo class II HLA-DSA	5.6% (3.1 – 8.1)	12.1% (8.4 – 15.7)	16.1% (11.7 – 20.4)

Fig. S2. Cumulative incidence of d*e novo* HLA-DSA at 1, 3 and 5 years after transplantation in 273 patients with first kidney transplant, without pre-transplant HLA-DSA and without desensitisation protocols. **Panel a**, data reported by the respective transplant centers according to the center-specific cut-off for HLA-DSA positivity; **panel b**, data calculated using a uniform MFI cut-off value of 1400.

a

	**1-year incidence** (95% CI)	**3-year incidence** (95% CI)	**5-year incidence** (95% CI)
de novo class I HLA-DSA	3.0% (0.9 – 5.0)	5.1% (2.3 – 7.8)	10.1% (5.9 – 14.1)
de novo class II HLA-DSA	5.7% (2.9 – 8.5)	18.3% (13.2 – 23.1)	27.7% (21.4 – 33.5)

b

	**1-year incidence** (95% CI)	**3-year incidence** (95% CI)	**5-year incidence** (95% CI)
de novo class I HLA-DSA	2.6% (0.7 – 4.5)	3.9% (1.5 – 6.2)	7.2% (3.7 – 10.7)
de novo class II HLA-DSA	4.1% (1.7 – 6.4)	12.6% (8.3 – 16.6)	17.5% (12.3 – 22.3)

Fig. S3. Cumulative incidence of ABMR subtypes during the first 5 years posttransplant in 273 patients with first kidney transplant, without pre-transplant HLA-DSA and without desensitisation protocols.

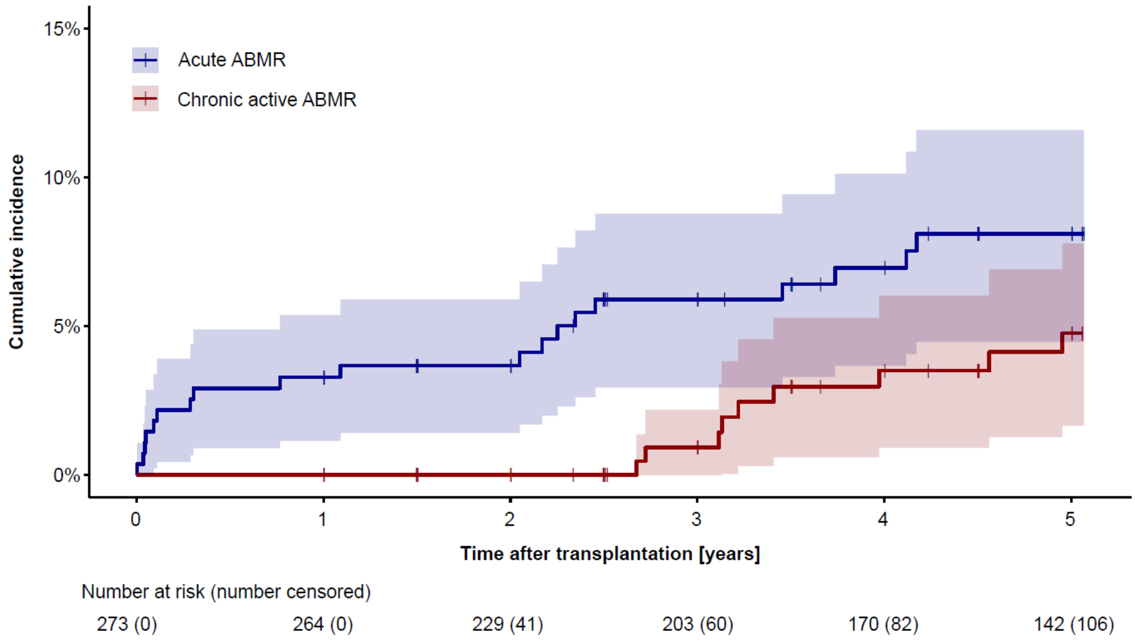

	1-year incidence (95% CI)	3-year incidence (95% CI)	5-year incidence (95% CI)
**Acute ABMR**	3.3% (1.2 – 5.4)	5.9% (2.9 – 8.8)	8.1% (4.5 – 11.6)
**Chronic active ABMR**	0% (0 – 0)	0.9% (0 – 2.2)	4.8% (1.7 – 7.8)
